# The Vivid Present: Visualization Abilities Are Associated with Steep Discounting of Future Rewards

**DOI:** 10.3389/fpsyg.2017.00289

**Published:** 2017-03-06

**Authors:** Trishala Parthasarathi, Mairead H. McConnell, Jeffrey Luery, Joseph W. Kable

**Affiliations:** ^1^Neuroscience Graduate Group, Department of Neuroscience, University of Pennsylvania, PhiladelphiaPA, USA; ^2^Department of Psychology, University of Arizona, TucsonAZ, USA; ^3^Department of Psychology, University of Pennsylvania, PhiladelphiaPA, USA

**Keywords:** delay discounting, visualization, future thinking, intertemporal choice, imagination

## Abstract

Humans and other animals discount the value of future rewards, a phenomenon known as delay discounting. Individuals vary widely in the extent to which they discount future rewards, and these tendencies have been associated with important life outcomes. Recent studies have demonstrated that imagining the future reduces subsequent discounting behavior, but no research to date has examined whether a similar principle applies at the trait level, and whether training visualization changes discounting. The current study examined if individual differences in visualization abilities are linked to individual differences in discounting and whether practicing visualization can change discounting behaviors in a lasting way. Participants (*n* = 48) completed the Vividness of Visual Imagery Questionnaire (VVIQ) and delay discounting task and then underwent a 4-week intervention consisting of visualization training (intervention) or relaxation training (control). Contrary to our hypotheses, participants who reported greater visualization abilities (lower scores) on the VVIQ were *higher* discounters. To further examine this relationship, an additional 106 participants completed the VVIQ and delay discounting task. In the total sample (*n* = 154), there was a significant negative correlation between VVIQ scores and discount rates, showing that individuals who are better visualizers are also higher discounters. Consistent with this relationship but again to our surprise, visualization training tended, albeit weakly, to *increase* discount rates, and those whose VVIQ decreased the most were those whose discount rates increased the most. These results suggest a novel association between visualization abilities and delay discounting.

## Introduction

Humans often make decisions that involve tradeoffs between immediate and delayed consequences. For example, smokers enjoy the immediate pleasure of smoking a cigarette even though they may understand the long-term consequence of continued use. The extent to which an individual rejects large rewards in the future to obtain smaller rewards available immediately is known as delay discounting (Kable and Glimcher, 2007). Humans and other animals frequently discount the delayed consequences of their actions (Chung and Herrnstein, 1967; Mazur, 1984; Rachlin et al., 1991; Frederick, 2003). A reward that is delayed has a reduced effect on behavior compared to the same reward provided immediately. In humans, delay discounting can be measured by giving people choices between immediate and delayed rewards and using these choices to estimate their discount rate, an index of the extent to which the value of delayed rewards is discounted relative to immediate ones. Discount rates vary widely across individuals (Frederick et al., 2002; Kable and Glimcher, 2007), but are remarkably stable across time within an individual (Baker et al., 2003; Ohmura et al., 2006; Kirby, 2009; Senecal et al., 2012). Higher discount rates (steeper discounting) are associated with a variety of maladaptive behaviors, including drug and alcohol abuse, smoking, and obesity (Kirby et al., 1999; Petry, 2001; Audrain-McGovern et al., 2004; Kirby and Petry, 2004; Madden and Bickel, 2010; MacKillop et al., 2011). High discount rates are also associated with poorer academic performance (Duckworth and Seligman, 2005; Kirby et al., 2005), a greater likelihood of mortgage default (Meier and Sprenger, 2012), and greater likelihood of divorce (Reimers et al., 2009). Given these associations between steep discounting and important life outcomes, there is keen interest in understanding the psychological processes that drive individual differences in discounting, and in developing interventions that could impact discounting in a lasting way.

One process that could account for individual differences in discounting is an individual’s ability to vividly imagine future outcomes. Some theoretical accounts of discounting stress how delayed outcomes are less vivid or less concrete than immediate outcomes (Trope and Liberman, 2003; Rick and Loewenstein, 2008). Related computational models show how discounting could arise from a prospective process that mirrors retrospective memory – akin to a distant memory, an outcome in the far future is harder to bring to mind (Kurth-Nelson et al., 2012). While speculative, these process models could be linked to normative models that provide reasons for discounting future outcomes based on their uncertainty (Sozou, 1998; Redish and Kurth-Nelson, 2010), if vividness serves as a psychological cue for the certainty of a future outcome. Functional imaging studies support the notion that discount rates may depend on such prospective processes. Blood-oxygen level dependent (BOLD) signal in ventromedial prefrontal cortex (vmPFC), a region engaged when individuals are simply imagining the future, predicts individual discount rates (Ersner-Hershfield et al., 2008; Mitchell, 2009; Cooper et al., 2013). Specifically, lower discounters exhibited greater vmPFC activation when thinking about the far future, while higher discounters exhibited greater vmPFC activation when thinking about the near future (Cooper et al., 2013). In addition to these links to individual differences, recent studies have shown how engaging visualization processes can change discounting. Several studies have now demonstrated that asking people to call to mind a future event reduces the extent to which they discount a delayed reward in a subsequent choice, and the size of this effect is correlated with the vividness of the imagined event (Peters and Büchel, 2010; Benoit et al., 2011; Liu et al., 2013; Lin and Epstein, 2014).

However, the effects of imagining a future event on discounting that have been demonstrated to date are short-lived and do not seem to persist past the immediately subsequent choice. Whether there are more stable associations between the ability to imagine future events and discounting, and whether these abilities can be altered in a longer-lasting manner, is unknown. In the current study, we ask if individual differences in visualization are linked to individual differences in discounting, and whether these abilities and discounting can be changed in a lasting way after training in visualization. We hypothesized based on previous work that a greater ability to vividly imagine events would be associated with reduced discounting, and that visualization training would lead to a decrease in discount rates. Surprisingly, though, we found the opposite association, and furthermore practice with visualization tended to both increase the ability to vividly imagine and to increase discounting.

## Materials and Methods

### Visualization Training Intervention Experiment

#### Participants

Forty-eight paid volunteers (33 females, 15 males; mean age = 24.6 years, *SD* = 6.5 years) from the University of Pennsylvania community participated in this study examining the effects of visualization training on discounting. All participants were healthy adults without any physical and/or mental illnesses. Ten participants did not complete the training period and therefore did not return for a follow-up visit to complete the study. The mean age of the final sample (*N* = 38, 28 females, 10 males) was 24.7 years (*SD* = 6 years). All participants provided consent in accordance with the procedures of the Institutional Review Board of the University of Pennsylvania.

#### Tasks

All participants completed two testing sessions, an intake session before the intervention (range = 8–28 days, median = 18 days) and a follow-up after the intervention (range = 3–19 days, median = 7 days). At each visit, participants completed the same battery of decision-making tasks and self-report questionnaires. Before the intake session, participants were randomly placed into either the intervention group (visualization training) or the control group (relaxation training). The final sample consisted of 20 participants in the intervention group and 18 in the control group. At the end of the intake session, those in the intervention group were also asked to write down 4–6 goals that they hope to achieve in the future.

All participants completed five decision-making tasks, presented on a computer using E-Prime (Psychology Software Tools, Sharpsburg, PA, USA). Tasks were presented in a random order for each subject. Our *a priori* hypotheses concerned the inter-temporal choice (ITC) task. The ITC task consisted of 102 choices, adopted from Senecal et al. (2012), Experiment 3. Each choice was between a smaller monetary reward available immediately or a larger reward available after a delay. Amounts for smaller rewards ranged between $10–$34, and amounts for larger rewards were $25, $30, and $35. The delays ranged from 1 to 180 days. All participants were presented with the same choices in a random order.

Four additional decision-making measures were administered for exploratory purposes: risk aversion, in which participants chose between a smaller amount of money that was certain and a larger amount that was risky (50% chance of receiving the reward, Levy et al., 2009); loss aversion, in which participants chose whether to take a gamble with a 50% chance of winning some amount and a 50% chance of losing a larger or smaller amount (Tom et al., 2007); ambiguity aversion, in which participants chose between playing a lottery with a fixed 50% chance of winning and another lottery where the reward was greater but the probability of winning was uncertain (Levy et al., 2009); and a task that measures the balance of model-based vs. model-free reinforcement learning (Gläscher and Büchel, 2005; Otto et al., 2013). As these tasks do not bear on our *a priori* hypotheses, detailed results from these measures will not be included in this paper.

#### Questionnaires

Following the battery of decision-making tasks, six self-report questionnaires were administered to participants using Qualtrics Online Surveys. The Vividness of Visual Imagery Questionnaire (Marks, 1973) was of main interest. The VVIQ is a 16-item questionnaire that measures individual differences in vividness of visual imagery. The questionnaire instructs participants to imagine different scenarios and subsequently rate their imaginations on a 4-point scale. Studies have reported an internal consistency reliability of 0.96 for the VVIQ (Rossi, 1977; Richardson, 1994; McKelvie, 1995; Campos, 2011). The VVIQ was used to test for an association between vividly imagining events and delay discounting, and as a manipulation check to ensure that visualization training in fact affected the ability to vividly imagine events.

The remaining five questionnaires were exploratory to test for other possible effects of the visualization training on self-reported traits: the Attributional Style Questionnaire (Peterson et al., 1982), measuring optimistic and pessimistic explanatory styles; the General Self-Efficacy Scale (Schwarzer and Jerusalem, 1995), assessing perceived self-efficacy; the Life Orientation Test (revised) (Scheier et al., 1994), measuring dispositional optimism; the Zimbardo Time Perspective Inventory Questionnaire (Zimbardo and Boyd, 1999), assessing orientation and attitudes toward time; and the Interpersonal Reactivity Index (Davis, 1983), measuring dispositional empathy and perspective taking.

#### Training

The training period lasted 4 weeks. For both groups, the training consisted of several 1-h guided meditation sessions conducted in a group, as well as 5-min online podcasts participants could listen to on their own.

The 1-h in-person guided meditation sessions were held in the Meditation Room of the Graduate Student Lounge at the University of Pennsylvania. Each intervention group underwent separate meditation sessions, offered 2 days a week, one in the evening and one mid-day. All sessions were led by the same instructor, a Health and Wellness Educator at the University of Pennsylvania and an experienced meditation and mindfulness instructor. Participants were asked to complete at least six of the eight in-person sessions offered to their group.

The meditation sessions for both groups were 1 h long and began with the same relaxation cues. For the visualization training group, this was followed by goal-oriented guided visualization. Participants were told to focus on a goal that they would like to achieve in the future and were led through two vivid scenarios in which they could imagine overcoming the obstacles in their way and experiencing the feelings accompanying achievement of the goal. The meditation sessions for the control group consisted of guided relaxation, without visualization or future thinking. Participants were told to bring awareness to their body and breath, and focused on the physical sensations they were experiencing in the present moment. (See Supplementary Material for full-length scripts).

The online podcasts were 5-min voice recordings by the same instructor, designed to be a shortened version of the in-person sessions. Participants were asked to listen to the podcast at least six times a week during the training period.

#### Payment

Participants were paid a show-up fee of $15.00 for the intake visit and $10.00 for the follow-up. At both visits, participants were aware that they could also receive an additional incentive-based payment according to their choices in one of the five decision-making tasks.

Each decision making task was designed to be incentive compatible. At the end of each session, the participant rolled a die to choose which of the five tasks would determine their payment. With the exception of the learning task, which was paid based on total performance, participants rolled a die again to determine the choice within that task for which they would be paid. For the ITC task, participants were paid using a Ficentive gift card (Sunrise Banks N.A., St. Paul, MN, USA), which was loaded with their earnings either the same day if they chose the immediate option, or after the specified delay if they chose the later option. Any gambles selected were resolved by flipping a coin (risk aversion, loss aversion) or drawing a poker chip from an envelope (ambiguity aversion).

For the training, participants received $10.00 for every 1-h meditation session they attended and $1.00 for every 5-min audio podcast they listened to.

#### Data Analysis

Discount rates were estimated using a logistic regression model in MATLAB (Mathworks). Participants’ choice data were fit with the following logistic function using maximum likelihood estimation:

$$\begin{array}{c} P_{1}=\frac{1}{1+e^{-\beta (SV1-SV2)}},\;P_{2}=1-P_{1} \end{array}$$

where *P*_1_ refers to the probability that the subject chose option 1, and *P*_2_ refers to the probability that the subject chose option 2. SV1 and SV2 refer to the participant’s estimated subjective value of option 1 and option 2, and β is used as a scaling factor. The subjective value of the options were estimated using a hyperbolic function:

$$\begin{array}{c} SV=\frac{A}{1+kD} \end{array}$$

where *A* is the reward amount, *D* is the delay, and *k* is the participant’s discount rate parameter (Kable and Glimcher, 2007). Larger values of *k* indicate a greater degree of discounting future rewards. To account for the fact that discount rates are not normally distributed, all statistics were performed on the log-transformed discount rates.

Since subjects were randomized to group and discount rates are known to be stable over time, we planned to evaluate the effects of visualization training using (1) a between groups *t*-tests comparing the visualization and relaxation groups after training, and (2) a paired-sample *t*-test testing within subjects differences in the visualization group between intake and follow-up. We also performed a between groups *t*-tests comparing changes after training in the two groups, equivalent to evaluating the interaction term in a mixed ANOVA. Pearson correlation coefficients were computed to assess the relationship between discount rates and self-report measures.

### Additional Samples Assessing Individual Differences

To allow us to examine the relationship between VVIQ and discount rates in a larger sample, we collected these measures in two additional experiments. Another 106 paid volunteers from the University of Pennsylvania community were recruited as part of two different studies examining the effects of different manipulations on discount rates. The first study included 49 subjects (30 females, 19 males; mean age = 23.3 years, *SD* = 3.6 years), while the second study included 57 subjects (36 females, 21 males; mean age = 22.3 years, *SD* = 3.3 years). All participants provided consent in accordance with the procedures of the Institutional Review Board of the University of Pennsylvania.

In both studies, participants came in on the 1st day and completed the same ITC task (102 questions) and VVIQ as in the visualization training intervention experiment. While the first study had no additional tasks or questionnaires on that 1st day, participants in the second study completed four other self-reports including the Gratitude Questionnaire (McCullough, 2002), which measures individual differences in the level of gratitude; the Grit Scale (Duckworth et al., 2007), which measures individual differences in perseverance and passion toward long-term goals; the Brief Mood Introspection Scale (Mayer and Gaschke, 1988), which measures current mood; and the Life Orientation Test (revised) (Scheier et al., 1994). Participants in both studies continued on to a 2nd day that involved a test of the effects of different manipulations on discount rates, but those results are not discussed here.

Participants were paid $10.00 an hour and received an additional incentive-based payment according to their choices in the ITC task (as outlined above). Discount rates and VVIQ scores were calculated in the same manner as above.

## Results

**FIGURE 1:**
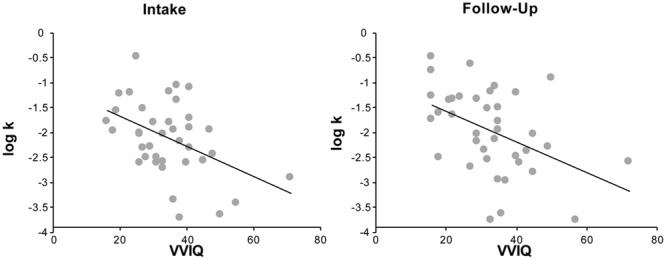
**Vividness of Visual Imagery Questionnaire (VVIQ) scores are significantly correlated with log-k values both at intake (*n* = 48, *r* = -0.37, *p* = 0.02) and at follow-up (*n* = 38, *r* = -0.45, *p* < 0.01)**.

**FIGURE 2:**
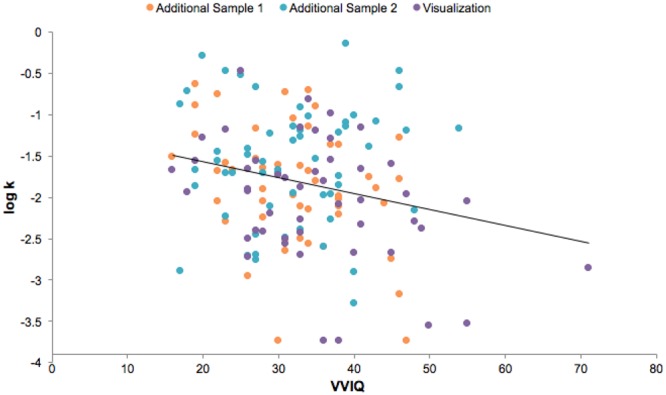
**Vividness of Visual Imagery Questionnaire (VVIQ) scores were significantly correlated with log-k values across all subjects in all experiments (*n* = 154, *r* = -0.25, *p* < 0.01)**.

Contrary to our hypothesis, we found that people who reported more vivid visual imagery were *higher* discounters. In the participants in our visualization training study, at baseline, VVIQ was significantly negatively correlated with log-k values (*n* = 48, *r* = -0.37, *p* = 0.02, note that lower VVIQ scores represent more vivid visual imagery, **Figure 1**). In other words, people with a greater capacity to imagine scenarios vividly on the VVIQ were less likely to select the larger delayed rewards in the intertemporal choice task. Since this result was in the opposite direction of our prediction, we collected VVIQ and discount rate data from additional subjects to evaluate the robustness of this relationship in a larger sample. Across all subjects (*n* = 154), the negative relationship between VVIQ and log-k values was attenuated but still statistically significant (*r* = -0.25, *p* < 0.01, **Figure 2**), indicating that individuals who were more vivid visualizers (lower VVIQ scores) were higher discounters (higher log-k values). This relationship persists when controlling for age, gender, and education [*R*^2^ = 0.09, *F*(4,149) = 3.737, *p* < 0.01, *b* = -0.209, *t*(149) = -2.599, *p* = 0.01]. Associations between other individual difference measures and discount rates or VVIQ are reported in **Table 1**.

**Table 1:** Relationship between inter-temporal choice (ITC) and Vividness of Visual Imagery Questionnaire (VVIQ) and all other tasks and self-reports administered in the visualization intervention experiment.

	Correlation with ITC pre-training	Correlation with VVIQ pre-training	Group differences post-training
**Tasks**	*n* = 48	*n* = 48	*n* = 38
Alpha, Risk Aversion	*r* = 0.27, *p* = 0.07	*r* = -0.19, *p* = 0.20	*t*(36) = 1.29, *p* = 0.21
Lambda, Loss Aversion	*r* = 0.00002, *p* = 0.99	*r* = -0.08, *p* = 0.59	*t*(36) = 0.54, *p* = 0.59
Beta, Ambiguity Aversion	*r* = 0.42, *p* < 0.01	*r* = -0.39, *p* < 0.01	*t*(36) = 0.61, *p* = 0.54
**Self-Reports**			
Life Optimism Test – Revised			
	*r* = -0.01, *p* = 0.95	*r* = -0.25, *p* = 0.09	*t*(36) = -0.88, *p* = 0.38
Zimbardo Time Perspective Inventory			
*Past Negative*	*r* = 0.10, *p* = 0.50	*r* = 0.10, *p* = 0.50	*t*(36) = -0.39, *p* = 0.70
*Present Hedonistic*	*r* = 0.13, *p* = 0.38	*r* = -0.27, *p* = 0.06	*t*(36) = 0.20, *p* = 0.84
*Future*	*r* = -0.001, *p* = 0.99	*r* = -0.07, *p* = 0.64	*t*(36) = 0.55, *p* = 0.58
*Past Positive*	*r* = -0.06, *p* = 0.71	*r* = -0.19, *p* = 0.20	*t*(36) = -1.16, *p* = 0.25
*Present Fatalistic*	*r* = 0.17, *p* = 0.25	*r* = -0.07, *p* = 0.64	*t*(36) = -0.45, *p* = 0.65
Interpersonal Reactivity Index			
*Perspective-Taking (PT)*	*r* = 0.41, *p* < 0.01	*r* = -0.45, *p* < 0.01	*t*(36) = 0.47, *p* = 0.64
*Fantasy (FS)*	*r* = 0.32, *p* = 0.03	*r* = -0.34, *p* = 0.02	*t*(36) = 0.09, *p* = 0.93
*Empathic Concern (EC)*	*r* = 0.22, *p* = 0.13	*r* = -0.21, *p* = 0.15	*t*(36) = -0.23, *p* = 0.82
*Personal Distress (PD)*	*r* = 0.30, *p* = 0.04	*r* = -0.13, *p* = 0.38	*t*(36) = 0.03, *p* = 0.97
Self Efficacy Scale			
	*r* = -0.007, *p* = 0.96	*r* = -0.24, *p* = 0.10	*t*(36) = -0.60, *p* = 0.55
Attributional Style Questionnaire			
*Good*	*r* = 0.09, *p* = 0.54	*r* = 0.10, *p* = 0.50	*t*(36) = 0.28, *p* = 0.78
*Bad*	*r* = -0.0001, *p* = 0.99	*r* = 0.02, *p* = 0.89	*t*(36) = -0.57, *p* = 0.57

*The first column shows the correlation with log-k values pre-training, the second column shows the correlation with VVIQ scores pre-training, and the third column tests for group differences between visualization and relaxation groups post-training.*

**FIGURE 3:**
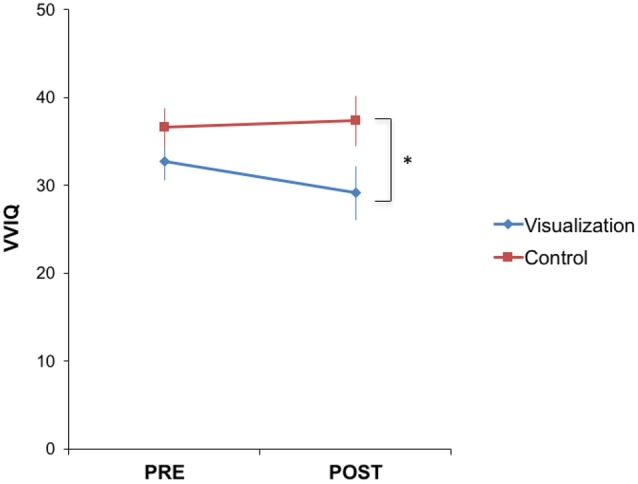
**Visualization training tended to decrease VVIQ.** The visualization group had significantly lower VVIQ scores (i.e., were more vivid visualizers) after training [*t*(36) = 2.20, *p* = 0.03], while there was no significant difference between the two groups before training [*t*(36) = 1.07, *p* = 0.29]. However, there was no significant effect of training within either group [*t*(19) = 1.54, *p* = 0.14 for visualization and *t*(17) = 0.81, *p* = 0.43 for control], and the interaction between group and time on VVIQ did not reach significance [*t*(36) = -1.66, *p* = 0.11]. ^∗^*p* < 0.05.

Returning to our visualization training study, we examined the effects of the visualization training on VVIQ scores. Consistent with our expectations, visualization training tended to increase the vividness of visual imagery, though this effect was not robust. After training, the visualization group (mean = 29.1, *SE* = 2.23) had significantly lower VVIQ scores than the control group (mean = 37.33, *SE* = 3.08) [*t*(36) = 2.20, *p* = 0.03, **Figure 3**]. Note that there was no significant difference between the intervention (mean = 32.75, *SE* = 2.18) and control (mean = 36.56, *SE* = 2.85) groups prior to training [*t*(36) = 1.07, *p* = 0.29]. However, there was no significant effect of training on VVIQ within the visualization group [*t*(19) = 1.54, *p* = 0.14; similar test in the control group, *t*(17) = 0.81, *p* = 0.43], and the interaction between group (intervention vs. control) and time (pre- vs. post-training) on VVIQ did not reach significance [*t*(36) = -1.66, *p* = 0.11].

**FIGURE 4:**
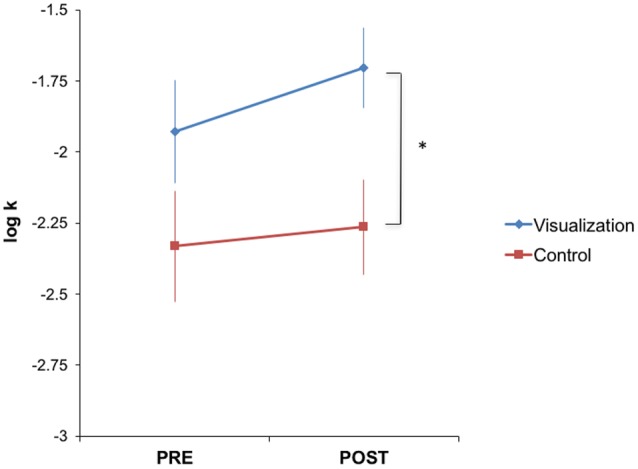
**Visualization training tended to increase discounting.** The visualization group had significantly higher discount rates (i.e., were more impatient) after training [*t*(36) = 2.16, *p* = 0.04], while there was no significant difference between the two groups before training [*t*(36) = 1.73, *p* = 0.09]. However, there was no significant effect of training within either group [*t*(19) = 1.83, *p* = 0.08 for visualization and *t*(17) = 1.04, *p* = 0.31 for control], and the interaction between group and time on discount rates did not reach significance [*t*(36) = 1.10, *p* = 0.28]. ^∗^*p* < 0.05.

We next examined the effects of visualization training on discounting. Contrary to our hypothesis, but consistent with the observed relationship between VVIQ and discount rates, visualization training tended to *increase* discount rates, though again this effect was not robust. After training, the visualization group (mean = -1.70, *SE* = 0.19) had significantly higher discount rates [*t*(36) = 2.16, *p* = 0.04] than the controls (mean = -2.26, *SE* = 0.17) (**Figure 4**). Note that there was no significant difference between the intervention (mean = -1.93, *SE* = 0.81) and control (mean = -2.33, *SE* = 0.60) groups prior to training [*t*(36) = 1.73, *p* = 0.09]. However, there was no significant effect of training within the visualization group [*t*(19) = 1.83, *p* = 0.08; similar test in control group, *t*(17) = 1.04, *p* = 0.31], and the interaction between group (intervention vs. control) and time (pre- vs. post-training) did not reach significance [*t*(36) = 1.10, *p* = 0.28]. Exploratory tests revealed no effects of training on any other measure (**Table 1**).

**FIGURE 5:**
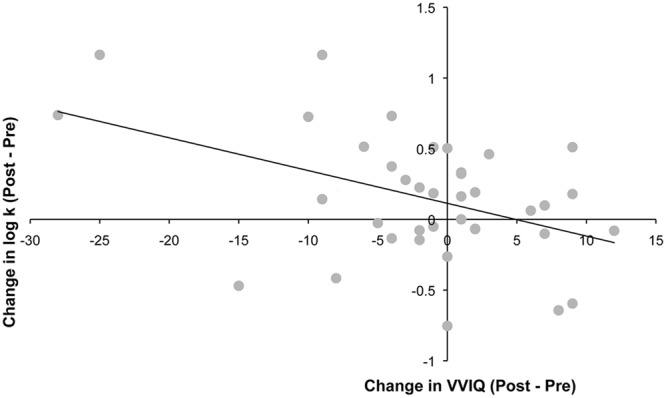
**Changes in log-k values after training were significantly correlated with changes in VVIQ scores (*r* = -0.44, *p* < 0.01)**.

Finally, we found that changes in discount rates after training were correlated with changes in the vividness of visual imagery. As at baseline, VVIQ and discount rates were negatively correlated after training (*n* = 38, *r* = -0.45, *p* < 0.01 at follow-up). Furthermore, there was a significant relationship between change in log discount rate from pre- to post-training and change in VVIQ (*r* = -0.44, *p* = 0.006). Individuals whose vividness of visual imagery increased the most were those whose discount rates increased the most (**Figure 5**).

## Discussion

Here we found that increased trait visualization abilities are associated with increased discount rates. In a total sample of 154 subjects, there was a significant negative correlation between VVIQ scores and discount rates, showing that individuals who are better visualizers are also higher discounters. Furthermore, consistent with this association between visualization abilities and discounting, we found that 1 month of repeated practice of visualizing one’s future goals tended, albeit weakly, to increase discounting of future rewards. After the intervention, participants in the visualization group had significantly higher discount rates than participants in the control group who performed relaxation exercises without visualization. Causal inference about the effect of the intervention, though, is weakened by the fact that the group by time interaction was not statistically significant. The intervention study did provide further correlational evidence for the relationship between visualization and discounting, as post-intervention changes in VVIQ were significantly negatively correlated with changes in discount rate. Taken as a whole, these findings provide converging support for the idea that the ability to vividly imagine scenes is associated with higher discount rates.

Given the growing evidence that instructing subjects to imagine future events leads them to discount less in subsequent choices (Peters and Büchel, 2010; Benoit et al., 2011; Liu et al., 2013), we had predicted the opposite association, that more vivid imagers would discount less and that visualization training would reduce discounting. Why might visualization abilities, as assessed by the VVIQ, be associated with steeper discounting?

One possibility is that since questions on the VVIQ ask subjects to visualize items in the present, the VVIQ specifically taps into the ability to visualize the present, perhaps at the expense of the future. If VVIQ measures visualization of the present, however, it is unclear why practice visualizing future goals would change VVIQ, as we observed.

A second possibility is that the ability to vividly imagine can be directed at the present and the future, and on balance yields further advantage for the already vivid present in tradeoffs between the two. Past research has shown that visualization in a concrete mindset raises levels of present-bias, which in turn increases impulsivity (Malkoc and Zauberman, 2006; Malkoc et al., 2010). Similarly, in classic experiments on delay of gratification, manipulations that increased the vividness of both outcomes (e.g., placing both rewards in front of the children) reduced delay of gratification and increased impulsivity (Mischel and Ebbesen, 1970).

A third possibility is that visualizing one’s future goals, in isolation, reduces rather than enhances one’s motivation toward those goals. Imagining achieving one’s future goals may serve as proxy for fulfilling those goals. In addition, visualizing goals that one has not yet reached, and the potential roadblocks to those goals, might have provoked anxiety and avoidance. Indeed, post-experiment feedback from some participants suggested that visualization training may have also enhanced visualization of the obstacles to achievement. This focus on goals that might not be achieved may lead to a sense of deprivation that promotes increased impulsivity (Hoch and Loewenstein, 1991; Rachlin and Raineri, 1992). These possibilities are further supported by the literature on *mental contrasting*, which shows that imaging future goals alone does not improve success in achieving those goals, unless accompanied by making concrete plans as to how to achieve those goals (i.e., *implementation intentions*) (Oettingen, 2000; Gollwitzer and Sheeran, 2006; Kappes et al., 2013; Oettingen et al., 2015).

Each of these three possible explanations for the trait associations we observed can be reconciled with reported state effects of imagination that go in the opposite direction, given that participants in studies of state effects imagine only future events, and these events are typically already planned or easily possible rather than highly desired goals (Peters and Büchel, 2010; Benoit et al., 2011; Lin and Epstein, 2014). Nonetheless, our results also lead us to reconsider potential explanations for why imagining future events has been shown to reduce discounting. In light of our results, it is possible that engaging in prospective thought or vivid imagination does not by itself drive these effects, but rather that imagining certain kinds of future events engenders positive emotions or reduces arousal that subsequently decreases discounting. This hypothesis is consistent with several studies regarding the influence of affect and arousal on discounting (Lerner et al., 2012; Kim and Zauberman, 2013), and would explain why in some cases imagining future events increases rather than decreases discounting. For example, Liu et al. (2013) found that participants were more likely to choose the immediate reward when imagining negative future events compared to no imagination; Lempert and Pizzagalli (2010) demonstrated that when a stressor is present, imagining an event in the future increases preferences for immediate reward; and Senecal et al. (2012) showed that engaging in prospective thought can, depending on the content of such thought, either increase or decrease discounting of delayed rewards.

Of course, it is also possible that our reported trait associations are not about imagination *per se*, but rather another personality trait associated with vivid visualization. For example, extraversion has been associated with lower VVIQ scores, with more extroverted individuals reporting more vivid imagination (McDougall and Pfeifer, 2012). It has also been reported that extroverted individuals are higher discounters (Ostaszewski, 1996; Hirsh et al., 2008, 2010), though not all studies find this (Becker et al., 2012; Manning et al., 2014), and reported associations are often moderated by other variables, such as cognitive ability (Hirsh et al., 2008) or current levels of positive mood (Hirsh et al., 2010). Since our study did not measure extraversion, it is possible that this trait could account for some of the relationship between visualization and discounting.

A few caveats to this study warrant mention. Both the training and the VVIQ assessment involved visualization activities that are fundamentally internal and subjective and thus difficult to verify. In addition, VVIQ scores could be affected by participants’ potentially faulty sense of how their own imagery compares to other people. Beyond this subjectivity, the podcast segment of the intervention was completed at home under conditions we were unable to monitor. Overall, we believe these considerations would have made it more difficult for us to detect effects of visualization training. We also cannot conclude that there are no favorable effects of visualization training. Though none of our exploratory variables showed any change, we measured a limited set of variables. We were also unable to assess whether participants in the visualization group were more successful in achieving their goals than they otherwise would have been.

Despite these caveats, our results provide novel evidence for an association between the vividness of visual imagery and discount rates. These results help to further delineate the complex relationship between episodic future thinking, vivid and concrete imagery, and delay discounting. Imagining possibilities more vividly may not always be the most productive path to increased patience.

## Ethics Statement

This study was carried out in accordance with the recommendations of the Institutional Review Board at the University of Pennsylvania with written informed consent from all subjects. All subjects gave written informed consent in accordance with the procedures of the Institutional Review Board. The protocol was approved by the Institutional Review Board at the University of Pennsylvania.

## Author Contributions

TP and JK designed the experiment and developed the analysis procedures. TP, JK, and MM wrote the paper. TP, MM, and JL collected and analyzed the data, discussed the results, and assisted in the editing process.

## Conflict of Interest Statement

The authors declare that the research was conducted in the absence of any commercial or financial relationships that could be construed as a potential conflict of interest.
