# Supplementary material for: The Vivid Present: Visualization Abilities Are Associated with Steep Discounting of Future Rewards
Source: Front Psychol. 2017 Mar 6;8:289. doi: 10.3389/fpsyg.2017.00289 (PMC5337487; doi:10.3389/fpsyg.2017.00289)
Supplement: Supplementary file 1 [file Data_Sheet_1.DOCX]

Supplementary Material

The vivid present: Visualization abilities are associated with steep discounting of future rewards

**Trishala Parthasarathi^*^, Mairead McConnell, Jeffrey Leury, Joseph W. Kable**

*** Correspondence:** Trishala Parthasarathi: trishala@mail.med.upenn.edu

# Supplementary Methods

**1.1 Visualization Group: Goal Oriented Visualization**

**1.1.1 First Scenario**

Take a moment to get comfortable and well supported in your seat. Become aware of all the many parts of your body that are touching your seat and touching the floor.

Gently close your eyes. Allow yourself to take up space and feel safe. (Pause)

Sit in an upright and relaxed posture, allowing each part of your body to feel connected. Attune to the breath as it moves in and out of your body. Feel supported by the earth underneath you. (*Sound of singing bowl)*

Begin to become aware of the rise of your belly as you breathe in and then notice as it settles back down as you breathe out. Bring your attention to the movement of the breath and the belly, allowing that to be your focus. There will be many times that your mind will wander, and you will begin to have conversations with yourself. When you notice this happening, very kindly and without any judgment, become aware of your thoughts or feelings and then let them go or let them be, and come back to the belly and the breath over and over again. This process is very natural and will happen many times during this guided meditation.

As you are breathing in and out, imagine that you are sending the warmth of your breath to any place in your body that is tense, tight or uncomfortable. Allow the warmth of the breath to loosen and relax all of the parts of your body from the top of your head, to the bottoms of your feet. (Pause) As you exhale, you may become more at ease and wish to release tension from your body.

The sound of my voice now will guide you into imagining a place where you can be safe, and comfortable, visualizing a goal that you would like to achieve.

Imagine you are sitting on a bench by yourself in a very peaceful spot. No one is around. You notice: there is a small blue bottle on the bench, immediately to your left. You are enjoying the day, and take in the quiet. It soothes you.

Now take a moment, and identify a goal that is important to you, a goal that you have not yet achieved, but that you truly desire. (Pause) See if you can visualize this goal that you have for yourself. (Pause) Breathe deeply and let the sensation of the goal move slowly through your body; from the top of your head to the bottom of your feet. Allow yourself to experience the goal in your body and in your mind. (Pause)

Now that you have identified this important goal, begin to think about the main obstacle that currently prevents you from reaching the goal. (Pause) Envision the obstacle, and notice how your body feels when you think about it. What emotions, if any, does envisioning this goal bring up for you? (Pause)

Imagine the obstacle as having a specific color, and a specific shape. (Pause) Take your time… You might want to enlarge the obstacle and make its color even brighter. (Pause)

Now pick up the blue bottle. You will notice that the liquid inside the bottle has a silver color. There are instructions on the label. They read: Place three drops on your obstacle, say goodbye, and then watch your obstacle shrink and disappear. (Pause)

You are now ready to address your obstacle. Pick up the bottle. Take out the dropper, place it above your obstacle, and squeeze the rubber tip...one drop falls on the obstacle, then a second, then a third.

Now say goodbye to the obstacle, as you close the bottle and set it down. (Pause) You may even notice some emotions connected to bidding farewell to that one significant thing that has prevented you from achieving your goal.

You hear a fizzing sound, as the drops hit the obstacle. As you watch, the obstacle slowly begins to shrink in size. Now it is three-quarters of its original size, now only a half, now a quarter, now you can hardly see it, as it totally disappears, and there is nothing there. (Pause)

The obstacle to your goal has completely disappeared. Really take that in. (Pause) How do you feel? (Pause)

Imagine now that you are waking up to a new morning without the obstacle. What is your first thought? (Pause) How are you feeling? (Pause)

You get up from your bed, and begin your day. You are now free to pursue your desired goal. How long do you think it might take to reach that goal, now that your main obstacle has disappeared? Will it take you a few days? (Pause) Will it take you a few months? Will it take you a few years to accomplish your goal? (Pause)

With your next breaths, the time needed to achieve your goal magically passes. (Pause)

Now imagine that you have achieved your goal, and you awaken on a typical morning. How does it feel to open your eyes to your life with this goal achieved? (Pause)

Now that your goal has been reached, think about how your life has been affected. (Pause) What do you see that lets you know that you have achieved your goal? (Pause) What do you hear? (Pause) What do you feel that lets you know that you have achieved your goal?

Imagine a particular time and place where you stand, having achieved your goal. Where is that scene located? (Pause)

In that scene, you look out through your own eyes …what do you see? Focus on the scene…what colors, do you see? (Pause) What shapes? (Pause) Sharpen your focus. What is immediately in front of you? (Pause) What is in the distance? (Pause)

When you think of yourself, and what you have achieved, what do you feel? (Pause) Dwell for a moment in that feeling, really take it in. (Pause) Now what words might you say to yourself to congratulate your hard work and perseverance? (Pause)

Imagine that you are telling a friend of your accomplishment. (Pause) How does that person react to the news? (Pause) What words do they use to congratulate you? (Pause)

Now imagine even more time has passed since you have achieved your goal. How does it feel to be in the future with your goal completed more than six months ago? (Pause) Do you feel any difference in your body…in your mind? (Pause) Do you notice a different sense of confidence in yourself? (Pause)

As you look back, with your goal achieved so many months ago, you recall the main obstacle that once prevented you from achieving your goal. From this vantage point, how does this obstacle appear to you now? (Pause)

In a few moments, after you hear the sound of the singing bowl, you can slowly open your eyes. When you do, you will feel refreshed, and ready to pursue your goal. (Pause) (Sound of the singing bowl)

May you continue to pursue your goals, feeling supported and optimistic.

**1.1.2 Second Scenario**

Take a moment to get comfortable and well supported in your seat. Become aware of all the many parts of your body that are touching your seat and touching the floor.

Gently close your eyes. Allow yourself to take up space and feel safe. (Pause)

Sit in an upright and relaxed posture, allowing each part of your body to feel connected. Attune to the breath as it moves in and out of your body. Feel supported by the earth underneath you. (*Sound of singing bowl)*

Begin to become aware of the rise of your belly as you breathe in and then notice as it settles back down as you breathe out. Bring your attention to the movement of the breath and the belly, allowing that to be your focus. There will be many times that your mind will wander, and you will begin to have conversations with yourself. When you notice this happening, very kindly and without any judgment, become aware of your thoughts or feelings and then let them go or let them be, and come back to the belly and the breath over and over again. This process is very natural and will happen many times during this guided meditation.

As you are breathing in and out, imagine that you are sending the warmth of your breath to any place in your body that is tense, tight or uncomfortable. Allow the warmth of the breath to loosen and relax all of the parts of your body from the top of your head, to the bottoms of your feet. (Pause) As you exhale, you may become more at ease and wish to release tension from your body.

The sound of my voice will now guide you into imagining a place where you can be safe, and comfortable, visualizing a goal that you would like to achieve.

It is morning, and you have just taken a seat in front of your computer.  You push the “on” key, and you hear the familiar sound of the computer booting up.

You are watching the monitor, as the computer goes through its usual processes.  The sign-in page appears on your screen.  At first glance, nothing seems unusual.  You see that your cursor is blinking in the space labeled “username,” and you type in your last name, as you always do.

You then move the cursor to the space immediately below, and prepare to type in your password.

However, to your surprise, the space that usually reads “password” now requests something different. It reads: “Name your goal.”

Now take a moment, and identify another goal that is important to you, one that is different than the goal that you identified in the first visualization. (Pause) See if you can visualize this new goal that you have for yourself. (Pause) Breathe deeply and let the sensation of the goal move slowly through your body; from the top of your head to the bottom of your feet. Allow yourself to experience the goal in your body and in your mind.  (Pause)

Once you have identified and experienced this new goal, imagine that you are typing a brief description of this goal, in the space provided. (Pause) Now imagine that you hit the “enter” key.

After you hit the “enter” key, another space appears, with the following request: “Name your greatest obstacle to achieving this goal.’

Now take a moment to identify the main obstacle that currently prevents you from reaching the goal. Envision the obstacle; what is it?  (Pause) Notice how your body feels when you think about it.  (Pause) What emotions, if any, does envisioning this obstacle bring up for you?   (Pause)

Now imagine typing into the space a brief description of your most important obstacle to achieving your goal. (Pause) You again hit the “enter” key, and now a picture of that greatest obstacle appears on the screen before you.  What do you see?  (Pause)

 Imagine the obstacle as having a specific color, and a specific shape. Take your time. (Pause) Allow your eyes to scan the picture of your greatest obstacle and notice its detail. (Pause)

 It is now time to confront this obstacle.  You move your cursor to the center of the screen, so it is directly on top of the obstacle. You then hit the “delete” key on your keyboard. Immediately, on your screen, there appears a message that reads:  “You are about to permanently delete your greatest obstacle.  Are you sure?” You think for a moment, and then you select “Yes,” and you click your mouse. (Pause)

There then appears on your screen a small red bar, which tracks the deleting of your obstacle.  You watch as the red bar slowly extends.  Your obstacle is 25% deleted; now the bar moves a bit more…it is 50% deleted….now 80%...and now 100%.  (Pause)

Say goodbye to your obstacle, as a new message appears on the screen. It reads: “Your obstacle has been permanently deleted.”   (Pause) The obstacle to achieving your goal is gone from your life.  Really take that in.  (Pause) You may even notice some emotions connected to bidding farewell to that one significant thing that has prevented you from achieving your goal.   (Pause)

 Imagine now that you are beginning your day, without the obstacle holding you back. What are your thoughts?  (Pause) How are you feeling?  (Pause)  You are now free to pursue your desired goal.  How long do you think it might take to reach that goal, now that your main obstacle is no longer there?  Will it take you a few days?  (Pause) Will it take you a few months? Will it take you a few years to accomplish your goal?  (Pause)

With your next breaths, the time needed to achieve your goal magically passes.   (Pause) Now imagine that you have achieved your goal, and you awaken on a typical morning. How does it feel to open your eyes to your life with this goal achieved?  (Pause)

Now that your goal has been reached, think about how your life has been affected. (Pause) What do you see that lets you know that you have achieved your goal? (Pause) What do you hear? (Pause) What do you feel that lets you know that you have achieved your goal?

Imagine a particular time and place where you stand, having achieved your goal.  Where is that scene located?   (Pause) In that scene, you look out through your own eyes …what do you see?  Focus on the scene…what colors, do you see?  (Pause) What shapes?  (Pause) Sharpen your focus.  What is immediately in front of you?  (Pause) What is in the distance?  (Pause)

When you think of yourself and what you have achieved, what do you feel?  (Pause) Dwell a moment in that feeling, really take it in.  (Pause) Now what words might you say to yourself to congratulate your hard work and perseverance?  (Pause)

Imagine that you are telling a friend of your accomplishment. How does that person react to the news?  (Pause) What words do they use to congratulate you?  (Pause)

Now imagine even more time has passed since you have achieved your goal.  How does it feel to be in the future with your goal completed more than six months ago? (Pause)  Do you feel any difference in your body…in your mind?  (Pause) Do you notice a different sense of confidence in yourself? (Pause) As you look back, with your goal achieved so many months ago, you recall the main obstacle that once prevented you from achieving your goal.  From this vantage point, how does this obstacle appear to you now?  (Pause)

In a few moments, after you hear the sound of the singing bowl, you can open your eyes.  When you do, you will feel refreshed, and ready to pursue your goal. (Pause) (Sound of the singing bowl) May you continue to pursue your goals, feeling supported and optimistic.

**1.2 Control Group: Relaxation Meditation**

**1.2.1 First Scenario**

Take a moment to get comfortable and well supported in your seat. Become aware of all the many parts of your body that are touching your seat and touching the floor.

Gently close your eyes. Allow yourself to take up space and feel safe. (Pause)

Sit in an upright and relaxed posture, allowing each part of your body to feel connected. Attune to the breath as it moves in and out of your body. Feel supported by the earth underneath you. (Sound of singing bowl)

Begin to become aware of the rise of your belly as you breathe in and then notice as it settles back down as you breathe out. Bring your attention to the movement of the breath and the belly, allowing that to be your focus. There will be many times that your mind will wander, and you will begin to have conversations with yourself. When you notice this happening, very kindly and without any judgment, become aware of your thoughts or feelings and then let them go or let them be, and come back to the belly and the breath over and over again. This process is very natural and will happen many times during this guided meditation.

Inhale fully and deeply...and release the breath slowly. Feel the sensation of letting go. (Pause) Inhale fully again, and exhale slowly. (Pause) One more time, inhale deeply and exhale slowly, feeling the sensation of letting go. (Pause) Now, allow the breath to go back to its natural rhythm. (Pause) Gently observe the breath, flowing in and out. (Pause)

You may begin to become aware of how observing the breath can bring you back to the present moment. Allow the awareness of your breath to move through your body. (Pause) Now, gently scan the various parts of your body. Are there areas that feel relaxed and at ease? (Pause) Are there any particular areas that feel tense, tight or uncomfortable? (Pause) Allow a gentle awareness to touch and relax those areas. (Pause)

Now bring your attention to the top of your head…then moving to the forehead, soften and relax from the inside out. (Pause) Move the warm energy of the breath to the eyebrows and then the eyes. (Pause) Imagine the eyes resting and feeling refreshed. (Pause) Now, using your mind and your breath, move down the face and soften the cheeks…the nose…and the jaw. (Pause) The jaw is a place in the body that you might hold emotions, tension or discomfort. Now, see if you can bring a slight smile to your face. A smile relaxes the muscles in the face and sends a message to the whole nervous system to relax and feel more at ease. (Pause) Continue to observe the breath. (Pause)

If you notice that the mind has drifted into thought….that’s perfectly natural. Just pause and notice where the mind has gone. Bring your attention to that place, acknowledge the thoughts, the emotions, or sensations, and then let them go, or let them be, and return to the breath. (Pause)

Now, move your attention to the neck. (Pause) Allow the breath to loosen the shoulders. (Pause) Follow the shoulders…down to the elbows…the elbows to the wrist…and open the palms. (Pause) Feel the sensation of an open and relaxed palm. (Pause) Rest in the present moment. (Pause)

Now using your breath and your attention, feel the chest and the back opening horizontally and vertically, creating space for the heart. (Pause) Allow the heart to open from the inside out. (Pause)

Now move your attention to the belly. Notice its rise, as you breathe in, and its fall, as you breathe out. (Pause) Feel the belly relax with each rise and fall. (Pause) Let the belly soften deep into the torso. (Pause)

Allow the body to be a safe place, a place where you feel at home. (Pause)

Now allow the warmth of your breath to move down the legs, touching your knees, your ankles, and then moving all the way down to your toes. (Pause) Feel the tension in the body being released through the soles of your feet. (Pause) Enjoy the feeling of letting go, and relaxing into your body. (Pause)

Imagine the softness of your breath infusing the body as a whole, from the top of your head to the soles of your feet. (Pause) The breath is now able to flow freely throughout the body. There may still be places in the body where tension or discomfort resides …that’s fine, let it be as it is. (Pause)

Now take note of what you are experiencing in your body and your mind. (Pause) If you are feeling more at ease, see if you can return to this feeling, as you go through your day, if only for a few breaths. (Pause)

Continue to follow your breath until you hear the sound of the singing bowl. (Pause) (Sound of singing bowl)

Follow the sound as it fades. (Pause) When you feel ready, slowly open your eyes. (Pause)

Without judgment, just notice how you are feeling. (Pause) Thank yourself for taking the time to relax in the present moment.

**1.2.2 Second Scenario**

Take a moment to get comfortable and well supported in your seat. Become aware of all the many parts of your body that are touching your seat and touching the floor.

Gently close your eyes. Allow yourself to take up space and feel safe. (Pause)

Sit in an upright and relaxed posture, allowing each part of your body to feel connected. Attune to the breath as it moves in and out of your body. Feel supported by the earth underneath you. (Sound of singing bowl)

Begin to become aware of the rise of your belly as you breathe in and then notice as it settles back down as you breathe out. Bring your attention to the movement of the breath and the belly, allowing that to be your focus. There will be many times that your mind will wander, and you will begin to have conversations with yourself. When you notice this happening, very kindly and without any judgment, become aware of your thoughts or feelings and then let them go or let them be, and come back to the belly and the breath over and over again. This process is very natural and will happen many times during this guided meditation.

As you breathe in, imagine that you are sending the warmth of your in-breath to any place in your body that is tense, tight or uncomfortable. (Pause) Allow the warmth of the in-breath to loosen and relax all of the parts of your body from the top of your head, to the bottoms of your feet. (Pause)

Now, focus for a moment on your out-breath. (Pause) As you exhale, you may become more at ease and wish to release tension from your body. (Pause)

Now, continue in a comfortable, upright and alert posture. Enjoy your breathing…in and out...allowing yourself an opportunity to arrive in the present moment. (Pause) Allow your arms to rest in your lap. (Pause) Allow your hands to connect with each other. (Pause) As each moment unfolds, allow a feeling of love to fill your heart… (Pause) …love toward yourself…and love toward all beings. (Pause)

As you continue the awareness of your breath you might begin to notice particularly its rhythm…Inhaling…exhaling. (Pause) Experience the vibration created by the breath, its quality and sound. (Pause)

Greet the in-breath and the out-breath with appreciation. (Pause)

Now notice the transition between the breathing in and breathing out. (Pause) Perhaps notice a pause between the in-breath and the out-breath, like a comma between words of a sentence. (Pause) Cultivate a kindly awareness of the present moment. (Pause)

If you find your mind wandering, gently notice where it is right now. (Pause) Perhaps it is focusing on what has already passed. (Pause) Or perhaps it is focusing on what has yet to be. (Pause) Gently return to the present moment by using the movements of your breath to help ground you. (Pause)

Softly smile and notice how that feels to you. (Pause) Smiling and breathing brings a reassurance that everything is ok as it is. (Pause)

Connect with your experience…moment by moment…by moment…keeping company with yourself in the present. (Pause) Notice again the pause between the in-breath and the out-breath… notice the silence of that pause. (Pause)

Observe what is happening in the body: what physical sensation are arising and passing? (Pause) Experience the movements of your breathing, sensations of the air being moved into the body and released. (Pause) Notice as your body fills as you breathe in, taking in nourishment for all the cells in your body. (Pause) Now, notice as the body empties as you breathe out, a sense of letting go, relaxing. (Pause)

Cultivate a sense of acceptance for whatever arises. (Pause)

Notice how breathing takes care of itself. Sit on the sidelines and just let your breathing happen. (Pause)

There is nowhere for you to go, and nothing to do. (Pause)

Now, as you breathe, you may feel compassion and acceptance for whatever you are experiencing in this moment. (Pause) Feel compassion and acceptance for any thoughts, feelings, or emotions that are present in this moment. (Pause)

As you go through your day, use your breath to bring you back to the present, to bring you back to a feeling of love and compassion for yourself and others. (Pause)

Thank yourself for taking this time to let go and be more in the present moment. (Pause)

Now, follow the sound of the singing bowl until it disappears. (Sound of singing bowl)

When you are ready, slowly open your eyes, and take time to bring yourself back to this room. (Pause)

As you go through your day, let each breath remind you of this meditation.
